# Kinetic modeling of ion formation during diesel combustion with different fuel injection timing

**DOI:** 10.1016/j.heliyon.2024.e28594

**Published:** 2024-03-22

**Authors:** Elaheh Neshat, Milad Mohammadi

**Affiliations:** Faculty of Mechanical Engineering, Sahand University of Technology, Sahand New Town, Tabriz, Iran

**Keywords:** Ion current, Diesel combustion, Injection timing, Multi zone model, Ionic mechanism

## Abstract

Ions are formed during the combustion process in internal combustion engines. The measurement of ions inside the combustion chamber produces reliable information about the combustion process. The present study focuses on the formation of ions inside the combustion chamber of diesel engines with different injection timing. For this purpose, a multi-zone thermodynamic model is utilized to simulate the closed cycle of the engine. To understand the kinetic behavior of the ions, the model is connected to an ionic chemical kinetics mechanism with 336 reactions and 81 species. Six important ionic reactions comprising 5 ions are used in the ionic mechanism. Dvode differential equation solver is also employed to calculate the energy and kinetics equations. The developed model has an acceptable accuracy in predicting the performance and pollutants of diesel engines. Based on the results, the ion formation is delayed by delaying the fuel injection timing. The maximum amount of in-cylinder ions depends on injection timing. In-cylinder ion current can predict the start of combustion accurately.

## 1 Introduction

Compression ignition (CI) engines are the most widely used type of internal combustion engines. They are utilized in transportation and various industries due to their high thermal efficiency. In these engines, air enters the combustion chamber, and at the end of the compression stroke, the fuel is injected into the combustion chamber [[1], [2], [3], [4], [5], [6]]. Many studies show that the injection time of the fuel has a significant effect on engine performance and emissions. Da Silva et al. proposed the effects of fuel injection timing on the performance of a diesel engine fueled with blended fuel of natural gas and diesel fuel [7]. Fan et al. studied the influence of injection timing and fuel injection angle on combustion performance using a 3D model and considering different fuel compositions [8]. Jiaqiang et al. assessed the effect of different injection timing and fuel injection pressure under different operating conditions on a biodiesel fueled CI engine [9]. Ashok et al. [10], Gong et al. [11], Natarajan et al. [12], and Deep et al. [13] examined the effects of fuel injection timing on diesel engine operation with different fuels. They reported a significant change in the heat release, as well as the in-cylinder pressure, by retarding the fuel injection. These results suggest the prominent role of fuel injection timing in controlling the combustion process. Fayad et al. studied the effect of the injection timing on diesel engine output PM. According to the results, the injection timing had a significant effect on PM size and morphology [14]. Li et al. investigated the effect of the injection timing on the combustion, performance and exhaust emission of a diesel engine. The engine was fueled with blends of the n-butanol, diesel and ethanol. The results showed that injection timing affects engine outputs strongly [15].

Ion current has been widely considered by combustion scientists as a method of detecting the characteristics of the in-cylinder combustion. In a diesel engine, ions are produced during the combustion process [16]. These ions move in a particular direction under an electronic field depending on their charge. In the areas where the electric field is formed between the positive and negative electrodes of the ion sensor (in some studies, the spark plug is used instead of the ion sensor [[17], [18], [19]]), the ion current is formed. The power of this current is detected by the sensors. Ion sensing is a low cost and reliable technology to study the internal combustion engine [20,21]. The ion current contains valuable information about the combustion process, such as the time of the start of combustion (SOC) [22], accurate NO_X_ emission prediction [23], air-fuel ratio estimation [19], in-cylinder pressure changes [24], knock estimation [17], misfire prediction [25], and the combustion intensity [26].

Although the effect of injection timing on the performance and emissions of CI engines has been discussed in several studies, no study has been done on the effect of injection time on the ion current inside the chamber. Considering that the ion current inside the chamber indicates the type of combustion inside the chamber (normal, knock or misfire), knowing the effect of the injection time on the ion current can indicate the appropriate injection time for efficient engine performance with higher power and normal combustion. The most important innovation of the present study is to fill the aforementioned study gap and investigate the effect of injection time on the amount of ions produced in the combustion chamber using a novel ionic chemical mechanism. In this study, engine performance was simulated using a thermodynamic model, and the ion current produced at different injection timing was calculated, and the related results were reported.

## 2 Methodology

### 2.1 Multi-zone model

**Fig. 1:**
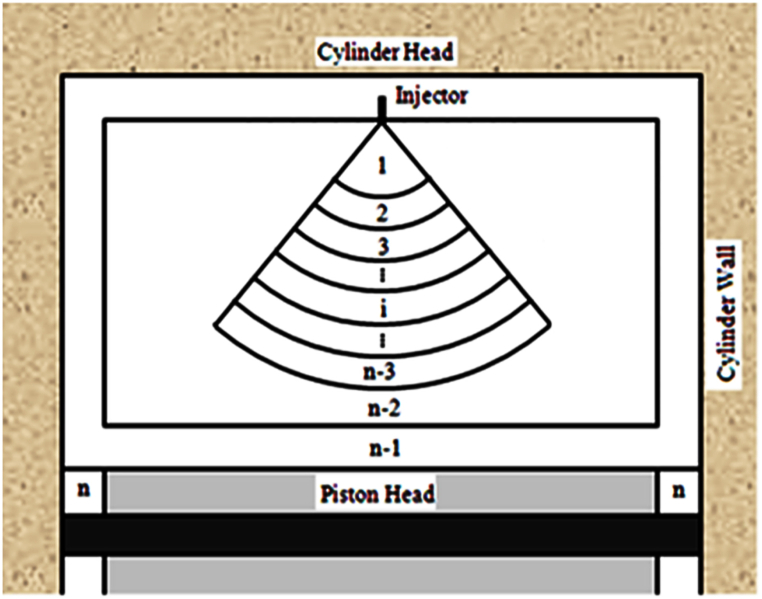
Zone configuration.

In this research, a multi-zone thermodynamic model is used to simulate the diesel engine performance. Figure (1) shows a schematic of the geometry of the multi-zone model.

In the mentioned model, the core, middle, boundary layer, and crevice zones are denoted by zone 1, zones 2 to n-2, zone n-1), and zone, respectively. The spray-cone angle was calculated utilizing Reitz and Bracco's model [27]. Energy and kinetics equations are solved simultaneously. Eqs (1), (2), (3), (4), (5) show the applied rules.

(1) $$\frac{{dU}_{i}}{dt}=\frac{{dm}_{in}}{dt}H_{in}-\frac{{dm}_{out}}{dt}H_{out}-\frac{{dW}_{i}}{dt}+\frac{dQ_{i}}{dt}$$

(2) $$\frac{{dU}_{i}}{dt}=c_{v}^{i}m_{i}\frac{{dT}_{i}}{dt}+m_{i}\sum_{j=1}^{n_{s}}u_{j}\frac{{dY}_{j\;}}{dt}+\sum_{j=1}^{n_{s}}u_{j}Y_{j}\frac{{dm}_{i}}{dt}$$

(3) $$\frac{{dW}_{i}}{dt}=P\frac{{dV}_{i}}{dt}$$

(4) $$\frac{dQ_{i}}{dt}=\frac{dQ_{i.cond}}{dt}+\frac{dQ_{i.conv}\;}{dt}+\frac{\;dQ_{i.rad}}{dt}$$

(5) $$\frac{{dY}_{k.i}}{dt}=\frac{{\dot{\omega }}_{k.i}{Mw}_{k}}{\rho_{i}}$$

The in-cylinder pressure is determined by Eq. (6).

(6) $$P=\frac{R_{u}\sum_{i=1}^{n_{z}}\frac{m_{i}}{{Mw}_{i}}}{\sum_{i=1}^{n_{z}}\frac{V_{i}}{T_{i}}}$$

**Fig. 2:**
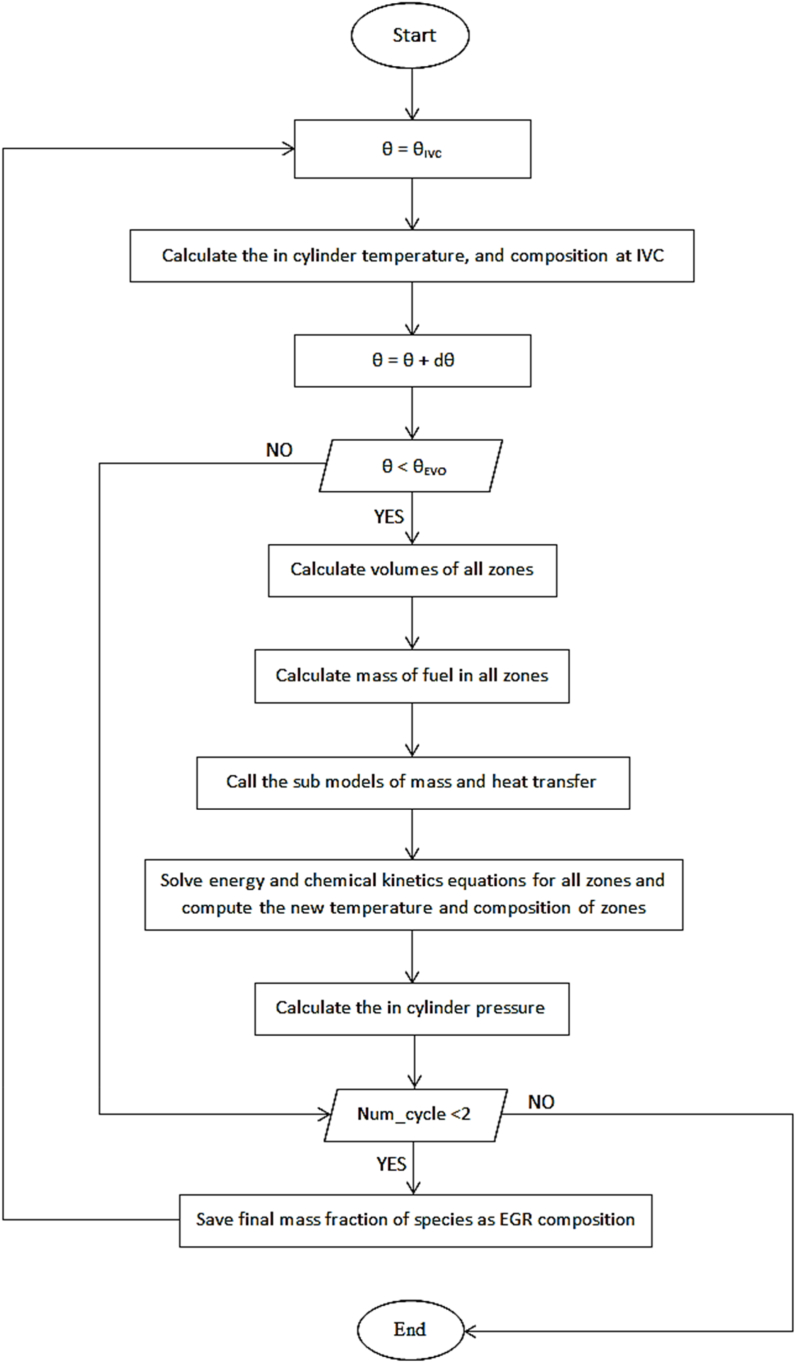
Problem solving algorithm.

Interested readers can refer to previous studies for more details of the equations governing the multi-zone model [28]. Figure (2) shows the problem solving algorithm. Modeling starts when the inlet valve is closed (IVC) and continued until the exhaust valve is opened (EVO). Initially, it is assumed that the mass and heat transfer between the zones are zero. The equations are solved using the DVODE differential equation solver. Then heat and mass transfer sub-models are activated, and the transferred energies are calculated. The mentioned equations are solved again and temperature and new composition are obtained. This process continues until all variables (species mass and temperature) converge. In the next step, the in-cylinder pressure is calculated. Bulk and diffusion mass transfer is used to increase the model's accuracy. It should be noted that the convergence accuracy in the present study is 10^−16^.

### 2.2 Ionic chemical mechanism

In the present study, the CHEMKIN subroutines are applied to simulate the combustion process. The mechanism has 330 reactions and 76 species [29]. This mechanism is related to C_14_H_28_ fuel. This fuel is converted to toluene and n-heptane in the chain initiation reaction, finally, it is converted to CH_2_O, HCO, CO and CO_2_. This mechanism consists of 6 and 14 reactions to simulate soot and NO_X_, respectively. An ionic mechanism with 6 reactions and 5 ionic species (Table (1)) is added to the main mechanism. The ionization inside the combustion chamber is finally analyzed using an ionic mechanism with 336 reactions and 81 species. The ionic mechanism is proposed in supplementary file.

### 2.3 Experimental data

**Fig. 3:**
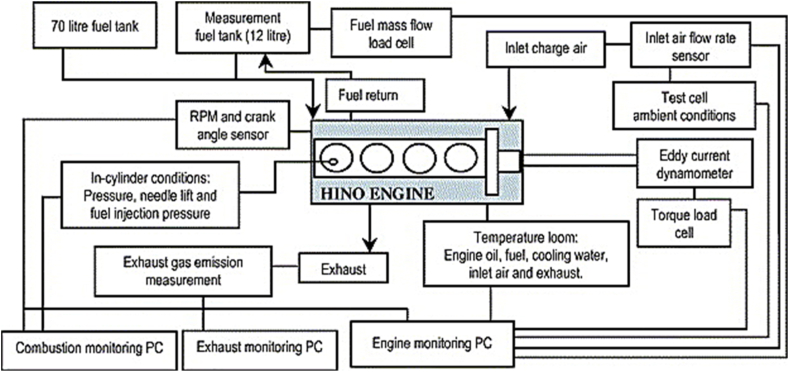
Experimental setup of the test engine.

HINO W04D engine experimental data are used for mechanism and multi zone model validation (see Table 1). The schematic diagram of the used setup is shown in Fig. 3. As it is clear in the figure, the engine has four-cylinders. This engine is four-stroke and direct injection and is connected to an eddy current dynamometer. The equipment used can create load and speed from 1 to 260 N/m and from 1 to 2400 rpm. The fuel flow meter also has an accuracy of 1 g. The engine inlet and outlet temperature is measured by two thermocouples. External gas analyzer can also measure NOx, CO, HC and CO_2_ pollutants with good accuracy. The accuracy of the HC and NOx measurement is as ±4 ppm and ±20 ppm [34]. The specifications of the engine are given in Table 2, Table 3, Table 4.

**Table 1:** Ionic reactions.

Number	Ionic reactions	**A (cm/mol s)**	β (−)	E_a_ (cal/mole)	Ref.
**1**	CH + O = e^−^ + HCO^+^	2.50E+11	0.0	7.09E+3	[30]
**2**	HCO^+^ + H_2_O=H_3_O^+^ + CO	1.00E+16	−0.1	0.00000	[31]
**3**	H_3_O^+^ + e^−^ = OH + H + H	7.95E+21	−1.4	0.00000	[32]
**4**	H_3_O^+^ + e^−^ = H_2_O + H	2.29E+18	−0.5	0.00000	[33]
**5**	OH + e^−^ = OH^−^	1.00E+17	−1.0	0.00000	[31]
**6**	e^−^ + O_2_ + N_2_=O_2_^−^ + N_2_	1.32E+17	−1.0	−8.30E+1	[23]

**Table 2:** Characteristics of HINO engine.

Engine type	Hino W04D
Displacement Volume (lit)	4.009
Bore (cm)	10.4
Stroke (cm)	11.8
Compression ratio	17.9
Number of nozzle holes	5

**Table 3:** Specifications of selected cases.

Case number	Rpm	Torque (Nm)	B. power (kw)	Air-Fuel ratio	Volumetric efficiency (%)	Air mass flow rate (kg/s)
A	1200	200	25.06	27.22	93.06	0.043
B	1600	130	21.81	42.63	93.28	0.056

**Table 4:** Numerical and experimental NO_X_ and Soot pollutants.

Case number	**Exp. Soot (**gr**)**	**Num. Soot (**gr**)**	**Exp. NO**_**X**_**(**gr**)**	**Num. NO**_**X**_**(**gr**)**
A	2.59E-5	5.05E-6	1.56E-3	5.58E-4
B	1.71E-5	3.67E-6	7.00E-4	1.43E-4

Two different operating conditions were considered to validate the production model whose details can be found in Table (3).

## 3 Results and discussions

### 3.1 Model validation

**Fig. 4:**
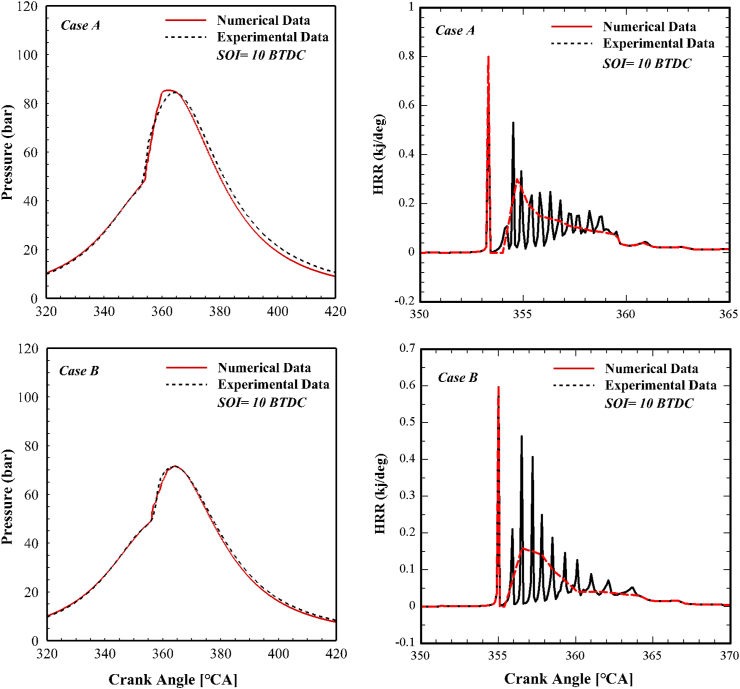
Experimental and numerical in-cylinder pressure and HRR traces.

In the first step, the thermodynamic model and coupled mechanism are validated against experimental data. The experimental and numerical pressure and the heat release rate (HRR) traces are shown in Figure (4). As seen, there is a good agreement between numerical and experimental data in the prediction of the SOC, CAD 50, and end of the combustion positions. HRR traces also indicate the high accuracy of the model and mechanism in predicting the performance of the diesel engine. Fluctuations in the HRR curve are due to the occurrence of combustion at different times in different places of the chamber. The maximum relative error in prediction of the peak pressure is 0.45%, in prediction of the SOC is 0.1%, and in the prediction of the position of the peak pressure 1.1%.

After examining the model capability in predicting engine performance, its capability should be evaluated in predicting emissions from diesel engines. Table (4) lists the experimental and numerical engine exhaust pollutants for the fuel injection start state in 10 BTDC. As can be seen, the model and mechanism managed to predict NO_X_ and Soot pollutants in both cases with acceptable accuracy.

### 3.2 Effects of fuel injection timing on engine performance

**Fig. 5:**
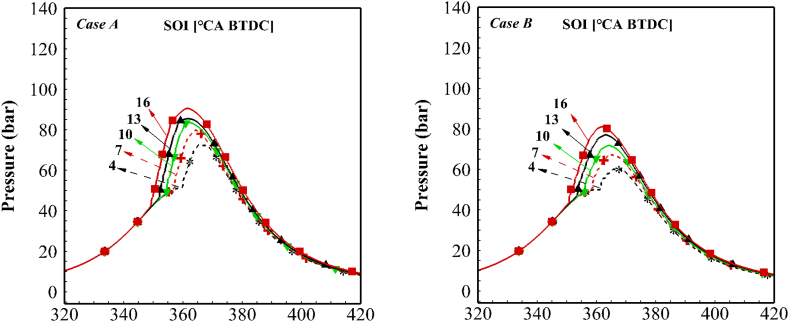
Effect of the injection timing on in-cylinder pressure for both of cases.

Figure (5) depicts the effect of the injection timing on the in-cylinder pressure in both cases. According to the figure, the retarded fuel injection reduces the maximum in-cylinder pressure due to lower ignition delay time, leading to less fuel evaporation before the premixed combustion. Therefore, the in-cylinder temperature and pressure of the compression stroke decreased near the top death center (TDC). The maximum pressure position was also delayed with retarding the injection time. Similar results were reported by other researchers [35,36].

**Fig. 6:**
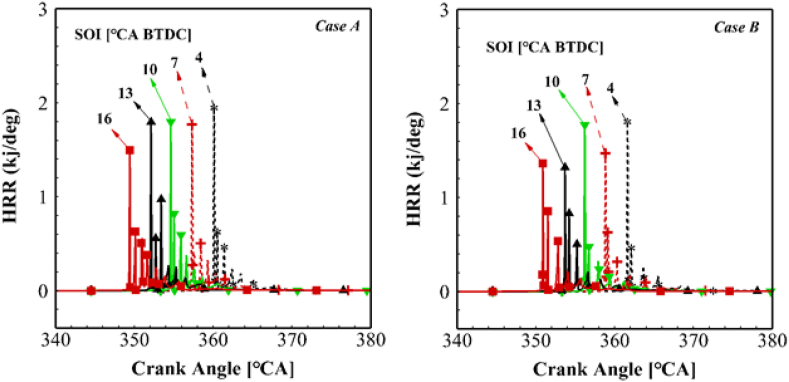
Effect of the injection timing on HRR for both of cases.

Figure (6) shows the effect of the injection timing on HRR in both cases. The figure shows that the peak of HRR in the core zone increases when injection timing retards, but the total heat release and peak value of the HRR in other zones is decreased. This phenomenon is because the temperature of the air inside the cylinder increases with the retarded injection of the fuel. This increase in temperature is higher in the core zone due to less heat transfer, and therefore combustion in the core zone takes place explosively. However in middle zones, due to lower air temperature and less ignition delay, the heat release rate is lower. The cumulative released heat decreases with retarded injection timing due to lower ignition delay and incomplete combustion.

**Fig. 7:**
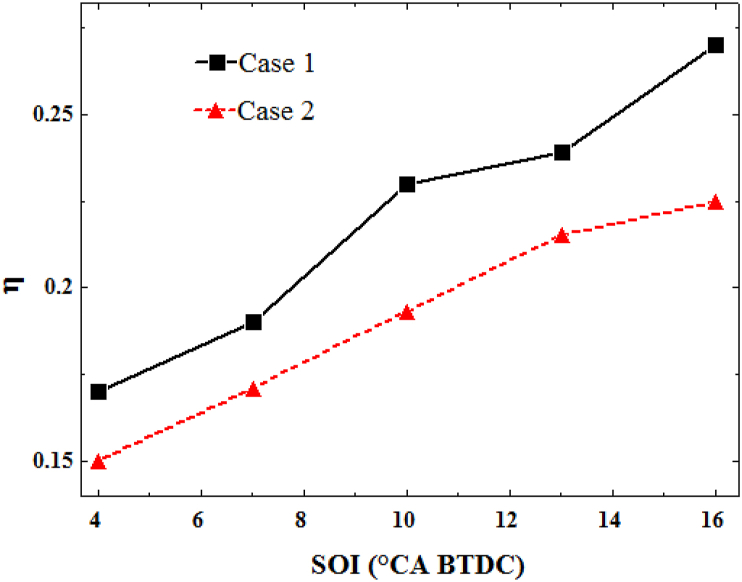
Effect of the injection timing on thermal efficiency for both of cases.

Figure (7) shows the effect of the injection timing on engine thermal efficiency. According to the figure, retarded injection decreases the engine output power and thermal efficiency. At the injection time of 16 BTDC, the highest amount of pressure and power is taken from the engine, hence, the highest engine performance is achieved at this injection time. Similar results were reported by Agarwal et al. [37].

### 3.3 The ability of ionic mechanism to predict ion current

**Fig. 8:**
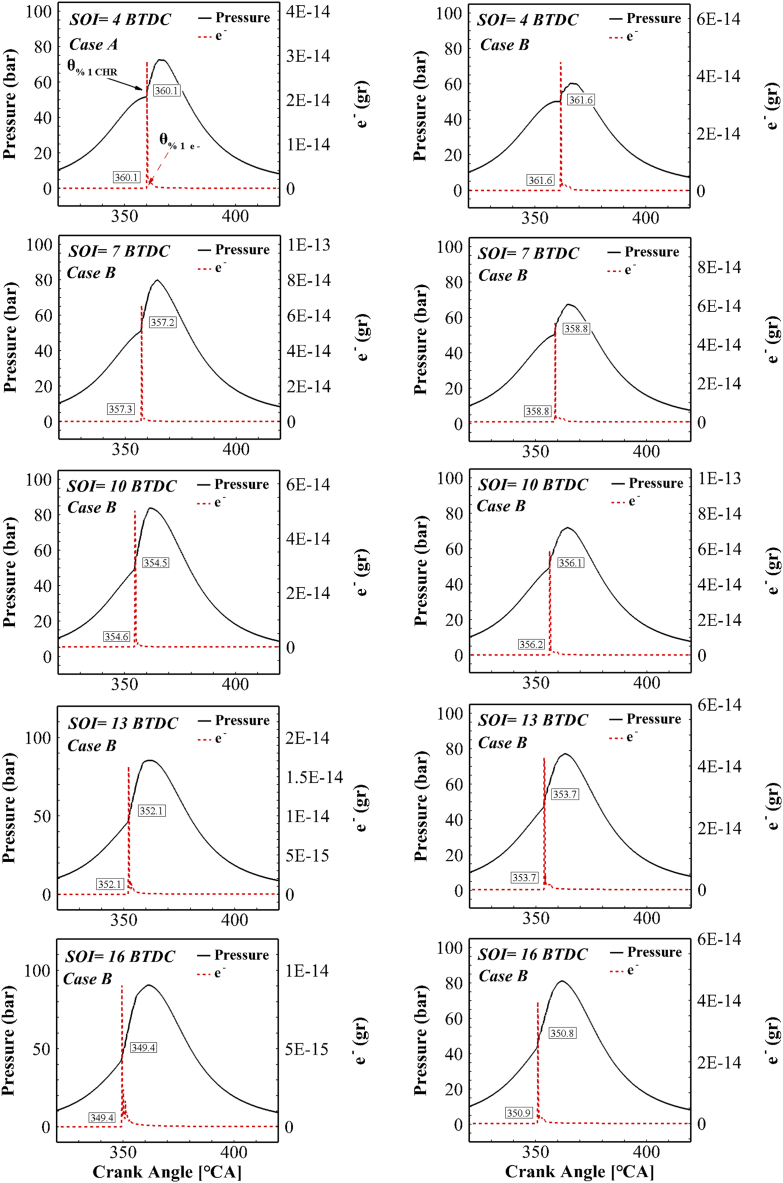
In-cylinder pressure curve with the generated electron in two different cases for different injection timing.

After predicting the performance and engine exhaust pollutants using simulation of combustion process chemistry, an ionic mechanism should be able to predict engine performance by estimating ion current in the combustion chamber. Upon combustion, chemical reactions are activated and the combustion process is controlled by radicals. Based on previous studies [38,39], combustion starts precisely where the electron is produced. Figure (8) depicts the in-cylinder pressure curve with the mass of electron generated relative to the crankshaft rotation angle for the previous cases for different injection timings. By delaying the fuel injection timing, the ignition timing is also delayed. By the retardation of the heat release time, the ion current start is also delayed. Sahin evaluated the issue of delaying the start of ion current by retarding the fuel injection time [40]. As a result, the start time of the electron at different injection conditions still coincided with the start time of combustion. Thus, there will be no difference in the accuracy of detecting the start time of combustion from the electron behavior by altering the fuel injection timing. Therefore, instead of expensive pressure converters, the combustion start time can be predicted at the desired accuracy under all different fuel injection conditions using cost-effective and efficient ion sensors.

**Fig. 9:**
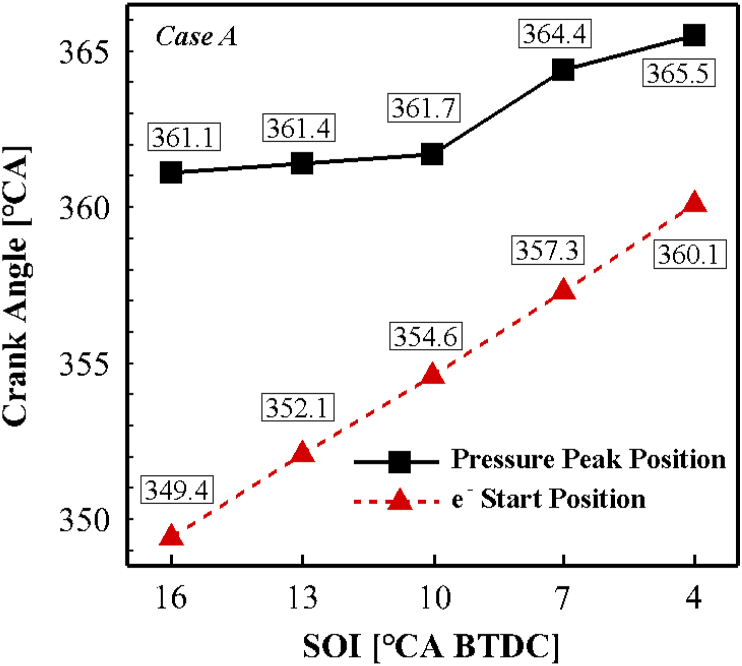
The changes of peak pressure position and start time of the electron formation for different fuel injection timings for case A.

Figure (9) shows the maximum position of the pressure and the start of the electron at different injection timing for case A. As seen, both the maximum position of in-cylinder pressure and the electron start time change by retarding the fuel injection start time, suggesting a significant relationship between pressure peak and electron production. Similar results were reported by Panousakis et al. [41].

**Fig. 10:**
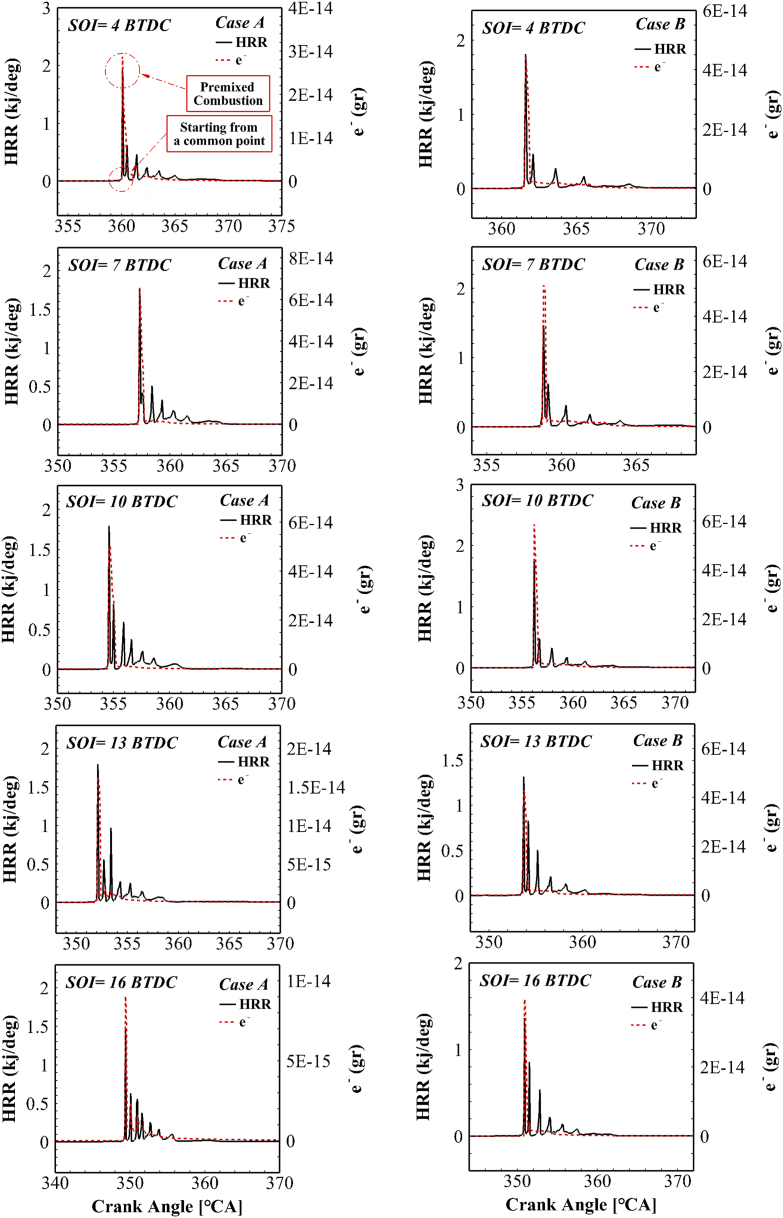
The electron mass and energy release rate in all different injection states for both cases.

Figure (10) presents the electron mass change curve and the energy release rate relative to the crankshaft rotation angle for different fuel injection timings. As can be observed, the production of electron inside the combustion chamber began with the start of premixed combustion. Moreover, the positions of the electron peak and the heat release rate peak coincided in the premixed combustion; this behavior remained stable with changes in operating conditions. Therefore, it can be said that there is a very strong correlation between electron and the energy release rate. Similar results were obtained by Attard and Micallef [42] for a controlled auto-ignition (CAI) engine. During diffusion combustion, lower chemical heat is released, and the mass of the electron is decreased. On the other hand, according to Figure (10), there is no change in the relationship between electron and HRR due to the changes in fuel injection timing.

### 3.4 In-cylinder ion current

**Fig. 11:**
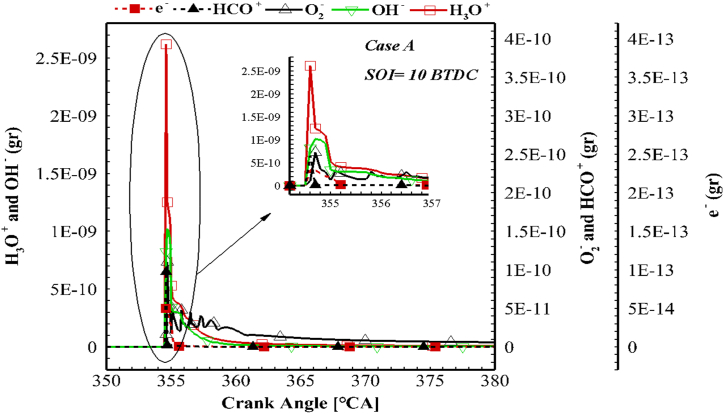
Mass variation of ionic species in case A for the injection time of 10 BTDC.

**Fig. 12:**
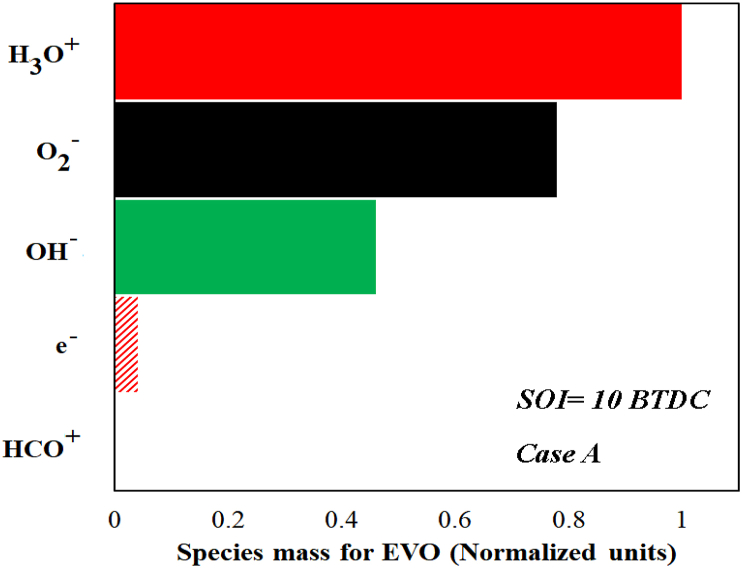
Mass of species at the EVO for case A and injection time 10 BTDC.

The ionic mechanism involves 3 negative ions (anions) and 2 positive ions (cations). The variations in the mass of all 5 ionic species for case A and the injection time of 10 BTDC are shown in Figure (11). H_3_O^+^ ion (hydronium) has the highest mass compared to other ions and was identified as the most important ion. This ion has also been reported as the main ion in several studies [20,43]. On the other hand, the electron has the lowest mass compared to other ionic species. Liu et al. reported similar results for the electron mass [44]. It should be noted that the electron is a very unique and productive species and plays a prominent role in the production of all negative ions. The H_3_O^+^ species and electron are not completely consumed after combustion (Figure (12)). Similar results were presented by Rao and Honnery [39]. The H_3_O^+^ ion has a significant mass in the end of in-cylinder combustion (as shown in Figure (12)). Using EGR, the ions enter the combustion chamber through the EGR system and the ionic reactions will be more active in the next cycle. Among the 5 introduced ions, only HCO^+^ ion was consumed completely. Similar results were reported by Aithal for propane fuel [43]. It was also found that the mass of OH^−^ ion is higher than O_2_^−^ as mentioned in previous studies [43,45].

**Fig. 13:**
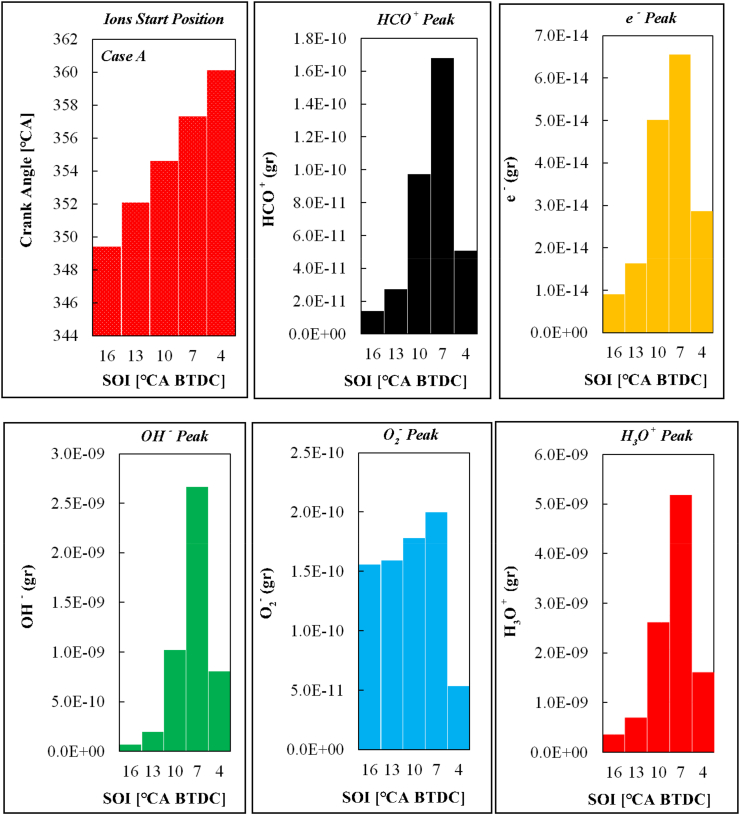
Start position curve and ions peak for all different injection timings related to case A.

Figure (13) depicts the starting position for all ions relative to the crankshaft rotation angle with the peak of each ionic species relative to the different fuel injection timing for case A. As can be seen, with the retardation of the fuel injection time, the ion start position is delayed. As mentioned earlier, the in-cylinder pressure peak was directly related to the starting position of the ionization current. As a result, ionization will be retarded by delaying the pressure peak.

**Fig. 14:**
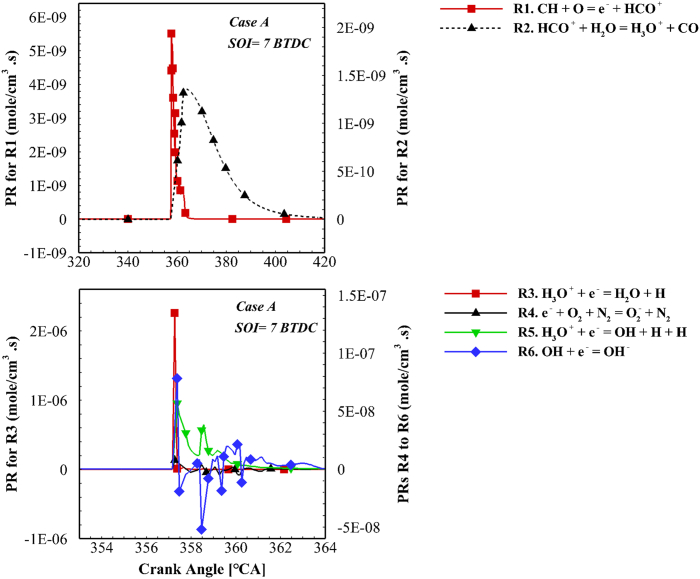
The PR curve of ionic reactions for case A and injection time of 7 BTDC.

According to Figure (13), injection timing has a significant effect on the ion formation. Most ions are produced at 7 BTDC and the lowest amount of ions are formed at 16 BTDC. Of course, O_2_^−^ is an exception whose behavior is slightly different from other species due to changes in injection timing. In a study by Sahin, a similar conclusion was reported [40]. The changes in the values of ionic species for different fuel injection timing can be assigned to the productive species in ion production during the combustion process. Figure (14) shows the progress rate (PR) of each ionic reaction for case A at the injection time of 7 BTDC. The PR of each reaction is obtained through Eq. (7) in FORTRAN software.

(7) $$PR_{i}=K_{f.i\;}\prod_{j=1}^{Ns}{\left[X_{j}\right]}^{\upsilon_{j.i}^{\prime }}\;‐\;K_{b.i\;}\prod_{j=1}^{Ns}{\left[X_{j}\right]}^{\upsilon_{j.i}^{\prime \prime }}$$

$\upsilon_{j.i}^{\prime }$ is the stoichiometric coefficient to the left of the reactions and $\upsilon_{j.i}^{\prime \prime }$ denotes the stoichiometric coefficient to the right of the reactions for the jth species corresponding to the ith reaction. In R1 (as shown in Figure (14)), a chain of fuel oxidation reactions is connected to a chain of ionic reactions. This reaction was the source of ion production in the ionic mechanism. Green and Sugden introduced this reaction as the ions source during the combustion [30]. In this reaction, CH and O are the productive species for ion production. The ion current gets stronger by raising the concentration of these radicals. Due to the lean combustion, the primary source of ions are oxygen radicals in diesel engines. The main source of oxygen radicals in the produced mechanism is the following reaction. This reaction proceeds in the backward direction and produces hydroxyl and O radicals by consuming O_2_ and H. Many studies have reported the following reaction as the first and most important reaction in the production of oxygen radicals [[46], [47], [48]].

$$O+OH\;↔O_{2}+H$$

This reaction is called the pool of radicals and is mainly aimed to provide O radicals for R1.

**Fig. 15:**
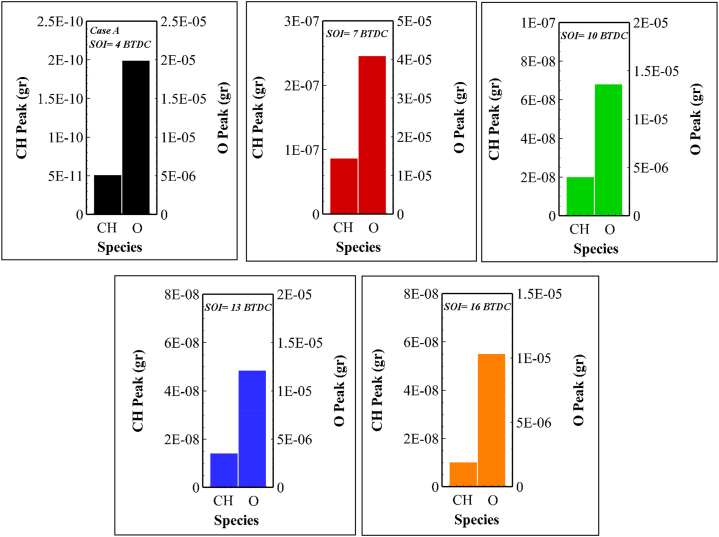
Peak values of CH and O for different fuel injection timings related to case A.

According to Figure (15), injection timing affects in-cylinder CH and O radicals. As mentioned earlier, the amount of O radicals is higher than CH in different injection timings. It is also observed that the highest amounts of CH and O are formed at 7 BTDC, explaining the increase in ionic species production (Figure (13)) in this case.

On the other hand, according to Figure (15), fewer electrons and HCO^+^ are formed in this state due to the weak mass of these two radicals in the injection state of 16 BTDC compared to other cases.

## 4 Conclusion

• This study investigates the effects of the injection timing on the ion formation in a diesel engine.
• A novel detailed chemical kinetics mechanism containing ions and ionic reactions is developed and used in the study.
• The obtained results show that for different injection times, the start time of the ion current coincides with the start time of the combustion. So, by using the ion current analysis, it is possible to accurately predict the start time of the combustion.
• The oxygen radicals are the primary source of ions. The OH^−^ is the most important ion; while electron is the least important species.
• The highest and fewest amounts of ions are produced at 7 BTDC and 16 BTDC, respectively.

## Data availability statement

Data will be made available on request.

## CRediT authorship contribution statement

**Elaheh Neshat:** Writing – review & editing, Writing – original draft, Validation, Supervision, Methodology, Investigation, Data curation, Conceptualization. **Milad Mohammadi:** Writing – original draft, Investigation, Formal analysis, Data curation.

## Declaration of competing interest

The authors declare that they have no known competing financial interests or personal relationships that could have appeared to influence the work reported in this paper.

## Nomenclature

Crank Angle Degree: *CAD*
Specific heat constant at constant volume (J/kg K): *c*_*v*_
Enthalpy (J/kg): *H*
Mass (kg): *m*
Number of zones: *n*
Number of species: *n*_*s*_
Pressure (Pa): *P*
Heat (J): *Q*
Universal ideal gas constant (J/mol K): *R*_*u*_
Temperature(K): *T*
Time (s): *t*
Internal energy (J): *U*
Volume (m^3^): *V*
Work (J): *W*
Mass fraction: *Y*

**Greek symbols**
$\dot{\omega }$: *Molar*rate of production (mole/m^3^s)
